# Different Immune Control of Gram-Positive and Gram-Negative Mammary Infections in Dairy Cows

**DOI:** 10.3390/vetsci11040166

**Published:** 2024-04-06

**Authors:** Giulio Curone, Joel Filipe, Alessia Inglesi, Valerio Bronzo, Claudia Pollera, Stefano Comazzi, Susanna Draghi, Renata Piccinini, Gianluca Ferlazzo, Alda Quattrone, Daniele Vigo, Massimo Amadori, Federica Riva

**Affiliations:** 1 Dipartimento di Medicina Veterinaria e Scienze Animali, University of Milan, 26900 Lodi, Italy; giulio.curone@unimi.it (G.C.); alessia.inglesi@unimi.it (A.I.); valerio.bronzo@unimi.it (V.B.); claudia.pollera@unimi.it (C.P.); stefano.comazzi@unimi.it (S.C.); susanna.draghi@unimi.it (S.D.); renata.piccinini@unimi.it (R.P.); alda.quattrone@unimi.it (A.Q.); daniele.vigo@unimi.it (D.V.); 2 Laboratorio di Malattie Infettive Degli Animali—MiLab, University of Milan, 26900 Lodi, Italy; 3 Pellegrina Extention Service, Veronesi Holding, 37142 Verona, Italy; gianluca.ferlazzo@gruppoveronesi.it; 4 Rete Nazionale di Immunologia Veterinaria, 25125 Brescia, Italy; m_amadori@fastwebnet.it

**Keywords:** mammary gland, dairy cow, mastitis, innate immune response, bacterial killing activity, NAGase

## Abstract

**Simple Summary:**

In the dairy industry, bovine mastitis poses significant challenges due to production losses and treatment costs. Understanding innate immunity in bovine milk could be crucial for fighting intra-mammary infections. We conducted cytofluorimetric analyses to assess bacterial killing activity in bovine skim milk against Gram-positive (*S. aureus*) and Gram-negative (*E. coli*) pathogens. We found that milk exhibited higher bacterial killing activity against *S. aureus* compared to *E. coli*, and those results correlated with N-acetyl-β-D-glucosaminidase (NAGase) levels. Both *S. aureus* killing and NAGase activity decreased with lactation duration. Milk samples from autochthonous Modenese cows displayed stronger killing activity compared to samples from cosmopolitan breed Holstein Friesian cows. Our findings suggest that skim milk is endued with distinct control mechanisms against different pathogens in the mammary gland, and provide insights into innate immune responses and potential strategies for managing mastitis in the dairy industry, which could lead to improved production efficiency and reduced treatment costs.

**Abstract:**

In the dairy industry, bovine mastitis represents a major concern due to substantial production losses and costs related to therapies and early culling. The mechanisms of susceptibility and effective response to intra-mammary infections are still poorly understood. Therefore, we investigated innate immunity in acellular bovine skim milk through cytofluorimetric analyses of bacterial killing activity against both Gram-positive and Gram-negative pathogens. Freshly cultured *E. coli* and *S. aureus* strains were incubated with colostrum and milk samples at different lactation time points from two groups of cows, purportedly representing mastitis-resistant and mastitis-susceptible breeds; bacterial cells were analyzed for vitality by flow cytometry following incorporation of vital dyes. N-acetyl-β-D-glucosaminidase (NAGase) activity was also investigated in milk and colostrum samples. Our findings revealed that colostrum and milk bacterial killing activity was greater against *S. aureus* compared to *E. coli*., with this activity correlated with milk NAGase levels. Furthermore, both killing of *S. aureus* and NAGase activity were negatively correlated to the elapsed time of lactation. Interestingly, samples from the allegedly mastitis-resistant breed displayed higher bacterial killing and NAGase activities. Our study suggests that diverse control mechanisms are exerted against Gram-positive and Gram-negative pathogens in the mammary glands of cows, probably beyond those already described in the literature.

## 1. Introduction

Mastitis remains a prevalent and economically burdensome disease in dairy farms. This inflammation of the udder leads to significant financial losses due to reduced milk production, milk disposal, premature culling of cows, and expenses related to veterinary care. Mastitis is primarily induced by bacterial intramammary infections (IMI), characterized by the influx of leukocytes and serum proteins from the bloodstream into the site of infection. Despite the adoption of updated management practices and genetic selection strategies, mastitis control remains challenging for the dairy industry [1]. Therefore, there is an ongoing research effort to understand both the pathogenesis of mastitis and the defensive mechanisms within the mammary gland [2,3]. Mastitis can be classified as either subclinical or clinical. Subclinical mastitis is characterized by intramammary infection without visible signs [4]. On the contrary, clinical mastitis is an inflammation that results in observable alterations in both milk and the affected udder. The severity of clinical mastitis ranges from mild to severe, with mild to moderate cases showing signs such as swelling, heat, pain, and redness in the udder, whereas severe cases exhibit symptoms like fever, anorexia, and shock [5]. The bovine mammary gland is endowed with several anatomical, physico-chemical, and immune-mediated defense mechanisms, including innate and adaptive immune responses. Bacteria entering the mammary gland encounter different anatomical barriers, such as epithelial cells equipped with Pattern Recognition Receptors (PRRs) that recognize them. The activation of PRRs triggers the transcription of genes encoding inflammatory molecules like cytokines, chemokines, and antibacterial factors [6]. Among PRRs, Toll-like Receptors (TLRs) are widely recognized [7]. They represent a group of highly conserved receptors that sense conserved motifs present in different pathogen microorganisms, known as Pathogen-associated Molecular Patterns (PAMPs) [8].

TLRs, after binding to their ligands, set off a signaling cascade that leads to NF-kB activation resulting in the expression of key response genes [9]. Bacterial infections trigger the recruitment of leukocytes to the udder via chemokines, with neutrophils (PMNs) showing the highest prevalence among infiltrating cells [10]. These cells eliminate bacteria through phagocytosis and the release of bactericidal molecules. Soluble molecules released by epithelial and leukocyte cells enrich milk with antimicrobial factors such as PTX3, lysozyme, lactoferrin, NAGase, defensins, cathelicidin, and complement proteins. The innate defenses of the mammary gland exhibit differences in response to Gram-negative and Gram-positive bacteria, as commonly observed in studies involving Holstein Friesian (HF) cows [2,11,12]. Gram-negative bacterial infections typically elicit a faster and more substantial innate immune response when compared to Gram-positive infections. In this regard, IMI caused by *E. coli*, a Gram-negative bacterium, and *S. aureus* and *S. uberis*, as Gram-positive models, have been extensively investigated for a long time. Gram-negative bacteria like *E. coli* are characterized by an immune active lipid A component in lipopolysaccharides (LPS). Together with lipopeptides, LPSs trigger NF-κB, AP-1, and TRF3 transcription factors, activating numerous genes. This stimulates mammary epithelial cells (MECs) to produce chemokines, antimicrobial peptides (AMPs), and macrophages to release TNF-α and IL-1β. In particular, IL-6 from MECs amplifies the self-defense mechanism by attracting circulating leukocytes, concurrently reducing milk secretion [13]. Gram-positive bacteria elicit a milder reaction, particularly in MECs. *S. aureus* carries lipopeptides, lipoteichoic acid, protein A, α-toxin, and other immunoreactive compounds. Yet, IL-1β and TNF-α production is much lower compared to *E. coli*-sustained IMI due to weaker NF-κB activation, whereas the AP-1 pathway is effectively stimulated, leading to IL-6 production [14]. This response also recruits leukocytes via chemokines, but self-defense activation is limited. Finally, in experimental IMI by *S. uberis*, more than 2200 genes are differently expressed compared to uninfected control quarters. Among these genes, there is a notable increase in the expression of immune-related genes (IL-1β, IL-6, TNF-α, TLR2, CXCL8, SAA3, lactoferrin, and complement C3), while the major milk protein genes show a decreased expression [15].

Moreover, an important element in the defense of the mammary gland against infections is the presence of antibacterial molecules in milk. The major antimicrobial molecules in milk are α-lactalbumin, β-lactoglobulin, lactoferrin, lysozyme, and various casein fractions. The concentration of these molecules is also influenced by the udder’s health condition. Indeed, several studies have demonstrated an increase in the levels of lysozyme and lactoferrin in mastitic milk [16,17]. Somatic cell count (SCC) is a widely used indicator for diagnosing subclinical mastitis and determining the selling price of milk. However, SCC may not always indicate udder infection due to other factors such as parity, lactation stage, milk production level, stress, season, and breed [18].

As for cattle breeds, some studies highlighted a lower susceptibility to mastitis of autochthonous cows compared to cosmopolitan dairy cattle [19,20]. Yet, clear correlates of mastitis resistance have not been defined. This is a major bottleneck in the development of new prophylactic tools and disease control strategies. Therefore, to gain a deeper understanding of milk bactericidal properties, we developed a novel assay on samples of cell-free skim milk; the assay was based on a user-friendly, flow cytometry approach toward possible large-scale usage, favorable costs, and discriminating power. Our experimental hypothesis was two-fold: (A) bactericidal and bacteriostatic properties are present in healthy, culture-negative bovine milk and may widely vary; (B) such preexisting properties are later modulated by the onset of IMI. The postulated variability of milk bacteriostatic and bactericidal activities prompted us to include in this study both allegedly mastitis-resistant, autochthonous Modenese (MOD) cattle and mastitis-susceptible Holstein Friesian (HF) cows, usually showing higher IMI prevalence under field conditions. Also, the use of two different breeds of cattle aimed to avoid a possible breed-related bias in our study.

HF is a well-known and characterized breed, whereas MOD cow, also called Bianca Val Padana, is an Italian native breed. It takes its name from the area of distribution around the provinces of Modena, Reggio Emilia, Mantova, Ferrara, and Bologna, where it was highly appreciated for its good quality milk. These versatile cattle serve dual purposes in milk and meat production, yielding on average 4700 kg of milk per lactation with 3.4% protein and 3.3% fat, which is ideal for Parmigiano-Reggiano cheese production [21]. The aim of our study was to verify the presence and the extent of bacterial killing activity in cell-free skim milk if this is modulated through the lactation period and/or by IMI, and if it is preferentially directed against Gram-positive or Gram-negative microorganisms.

## 2. Materials and Methods

### 2.1. Animals

Milk and colostrum samples were collected from two groups of cows, allegedly mastitis-resistant Modenese (MOD, 8 cows) and mastitis-susceptible HF (7 cows) breeds, under the supervision of experienced bovine practitioners. Cows were housed in the same farm in Parma province (Italy) with a free stall barn and milked using a pipeline milking system twice daily. Cows were fed ad libitum with a total mixed ration without silage using alfalfa, mixed grass hay, straw, and concentrated feed with mineral and vitamin supplementation. All cows had an average number of lactations between 1 and 5, with a mean of 2.3 for HF and 2.7 for MOD. Several lactation parameters were registered from each animal under study: daily milk production, lactation duration, and the content of proteins and fat in milk. This study complied with Italian and European laws on animal experimentation and ethics (Italian Health Ministry authorization no. 628/2016-PR). Production data of the animals were obtained from the analysis routinely carried out by the Italian Breeders’ Association.

### 2.2. Samples

The four-quarters milk and colostrum samples were collected from each animal at two different time points: 1 day after calving (T2) and 7–10 days after calving (T3). Quarter samples were kept separately over the whole study (no pooling at any time) because bacteriologically each quarter can be considered independent due to its anatomical structure [22]. Given that our study is the first one on this topic, we could not find any data in the literature to calculate the effect size and conduct a G*power analysis to calculate the adequate sample size. We then performed a post hoc analysis. We calculated the effect size based on our data by applying the formula of Sullivan et al. [23]. Finally, we calculated the statistical power of all our analysis using the G*power software version 3.1.9.4 (Franz Faul, Universitat Kiel, Germany) indicating the sample size used (total number of quarters, number of quarters at each time point) for each parameter: *S. aureus* killing activity, *E. coli* killing activity, and NAGase activity. All the analyses presented a statistical power higher than 80% (some of them higher than 90%) (Supplementary Figure S2). Four MOD and two HF cows were also sampled at dry-off (T1) and 30 days after calving (T4). Before milk and colostrum sampling, teat ends were carefully cleaned and disinfected with ethyl alcohol. First streams of foremilk were discarded, and then approximately 150 mL of colostrum/milk were collected aseptically from each quarter into sterile vials. Samples were delivered to the laboratory at 4 °C within 3 h and immediately processed. Two aliquots of whole colostrum/milk (10 mL each) were stored at −20 °C for bacteriological analysis and somatic cell count (SCC). Then, colostrum/milk (50 mL) was centrifuged for 10 min at 840× *g* at 4 °C. The fat layer (milk fat globules, MFG) and the cell pellet were discarded. This centrifugation removes epithelial cells and leukocytes, allowing the examination of soluble antibacterial compounds released in milk by these cells. For each sample, 1 mL of acellular skim colostrum/milk was transferred to a 1.5 mL sterile tube and immediately frozen at −80 °C for the bacterial killing and NAGase tests.

On the whole, we collected 60 quarter samples at both T2 and T3 and 24 quarter samples at both T1 and T4, which was in line with the expected range of statistical differences for the parameters under study.

### 2.3. Bacteriological Analysis and Somatic Cell Count

For bacteriological analyses, quarter colostrum/milk samples were thawed at room temperature. Ten µL of milk were plated onto 5% blood agar plates (Oxoid, Rodano, Milan, Italy). Plates were incubated under aerobic conditions at 37 °C and observed after 24 and 48 h. Bacteria were identified according to the guidelines of the National Mastitis Council [24]. Quarter somatic cell counts were determined using an automated counter (Bentley Somacount 150, Bentley Instrument, Chaska, MN, USA) based on laser flow cytometry.

### 2.4. Bacterial Killing Test

Two reference strains (one *S. aureus* and one *E. coli*) isolated from field clinical mas-titis samples, were amplified overnight at 37 °C in Brain Heart Infusion (BHI) medium. Then, the bacterial suspensions were diluted 1:20 in BHI and again grown at 37 °C for 2 h; the final concentrations of bacteria were determined by spectrophotometric analysis (BioPhotometer, Eppendorf, Hamburg, Germany). Each colostrum/milk sample was diluted 1:1 in BHI medium (0.25 + 0.25 mL). Twenty-five μL of the above-mentioned bacterial suspensions in the exponential phase of growth were added to each 1:1 colostrum/milk–BHI mixture in 1.5 mL sterile tubes (1:20 dilution), which were incubated at 37 °C for 2 h. Control samples included bacteria diluted 1:20 in the BHI medium (growth control) and uninoculated BHI medium. After the 2 h incubation period, samples were diluted 1:100 in sterile saline solution and immediately distributed in U-bottomed 96-well microtiter plates (200 μL/well). Bacterial viability was evaluated using the Live/Dead BacLight bacterial viability kit (Thermo Fisher Scientific, Waltham, MA, USA) for flow cytometry by adapting the manufacturer’s directions to microplate volumes. This kit is based on Cyto 9 and Propidium iodide, staining live and dead bacterial cells in the green and red fluorescence channels, respectively. Stained samples were analyzed before and after the 2 h incubation period by a Guava Easycyte HT flow cytometer (Merck Millipore, Burlington, MA, USA). After gating bacterial cells using forward and side scatter, bacterial viability was analyzed in a red x green cytogram, which allowed for discrimination between dead (gate R1; Supplementary Figure S1), live (gate R2; Supplementary Figure S1), and subvital cells (gate R4; Supplementary Figure S1). These gates were defined on the basis of two internal controls:

- Live cell control: Bacteria were reacted with 50% BHI in saline for 2 h at 37 °C, centrifuged at 10,400× *g* for 3 min at 4 °C, resuspended in the same volume of saline, and kept at room temperature for 60 min. Cells were again centrifuged, washed with sterile saline, and resuspended in 1 mL of saline.
- Dead cell control: Bacteria were reacted with BHI/saline for 2 h at 37 °C, centrifuged at 10,400× *g* for 3 min at 4 °C, resuspended in 70% isopropyl alcohol, and kept at room temperature for 60 min. Cells were again centrifuged, washed with sterile saline, and resuspended in 1 mL of saline.

The transition region between live and dead bacterial cells corresponded to subvital cells, showing intermediate permeability to propidium iodide.

Bacterial killing of colostrum/milk samples was calculated as the difference in dead/subvital cells post-incubation compared to pre-incubation. More in detail, the killing activity was expressed as the % sum of the events in R1 (dead) + R4 (subvital) regions (Supplementary Figure S1).

The bacterial cells of colostrum/milk microbiota of the samples were not a confounding factor in our assay, as shown in preliminary experiments on samples of non-inoculated, centrifuged skim milk [25].

### 2.5. NAGase Test

To assess NAGase activity, colostrum/milk samples were centrifuged at 16,000× *g* for 20 min, and then the enzyme activity was evaluated in duplicate using a fluorescence-based procedure, on a microplate fluorometer at 355 nm exc and 460 nm em (Fluoroskan Ascent, Thermo Fisher Scientific, Waltham, MA, USA), as previously described [26]. NAGase activity was expressed as Units (pmol of 4-methylumbelliferon released per min at 25 °C catalyzed by 10 μL milk).

### 2.6. Statistical Analysis

Statistical analyses were performed using SPSS 26.0 for Windows (IBM, Armonk, NY, USA), GraphPad Prism 6 (La Jolla, CA, USA), and PROC MIXED of SAS (version 9.3, SAS Institute Inc., Cary, NC, USA). For the statistical analysis of the bacteriological data, all quarter samples were included. The frequencies of different IMI-causing bacteria isolated in the two cattle groups were compared using a χ^2^ test. The risk of a sample being infected by a mammary pathogen was compared in two breeds by the Exact Fisher Test, and the odds ratio was subsequently calculated. The normality of SCC distribution values, coming from bacteriologically negative and positive samples, was assessed by the Shapiro–Wilk test. Since data were not normally distributed, different SCC values were compared by a nonparametric Mann–Whitney test. Differences in the bacterial killing and NAGase activities between the two groups (MOD and HF) at each time point were analyzed using Student’s *t*-test (for normally distributed data; Shapiro–Wilk test) or Mann–Whitney (for non-normally distributed data; Shapiro–Wilk test) test, considering statistically significant values of *p* < 0.05 and tendencies at *p* ≤ 0.10. Quarter samples were also divided into four categories according to the following criteria [27,28]:

- Negative: quarters with negative bacteriological analysis and SCC < 100,000;
- Inflamed: quarters with negative bacteriological analysis and SCC between 100,000 and 200,000;
- Latent: quarters with positive bacteriological analysis and SCC < 200,000;
- Subclinical: quarters with SCC > 200,000.

Given that only two-quarters belonged to the inflamed group, this group was not analyzed. Differences in the killing and NAGase activities among the three groups (negative, latent, and subclinical quarters) were analyzed using the Krustall–Wallis test (for non-normally distributed data; Shapiro–Wilk test), considering statistically significant values of *p* < 0.05 and tendencies at *p* ≤ 0.10. Differences in the killing and NAGase activities between the two groups (culture-negative quarters with SCC < 200,000 and culture-negative quarters with SCC > 200,000) were analyzed using the Mann–Whitney test (for non-normally distributed data; as shown by the Shapiro–Wilk test), considering statistically significant values at *p* < 0.05 and tendencies at *p* ≤ 0.10.

Finally, two-tailed rho tests of Spearman were used to reveal correlations among bacterial killing activity, NAGase activity, and all the other parameters under investigation.

## 3. Results

### 3.1. MOD Group Produced Less but Higher Quality Milk Compared to HF

**Figure 1:**
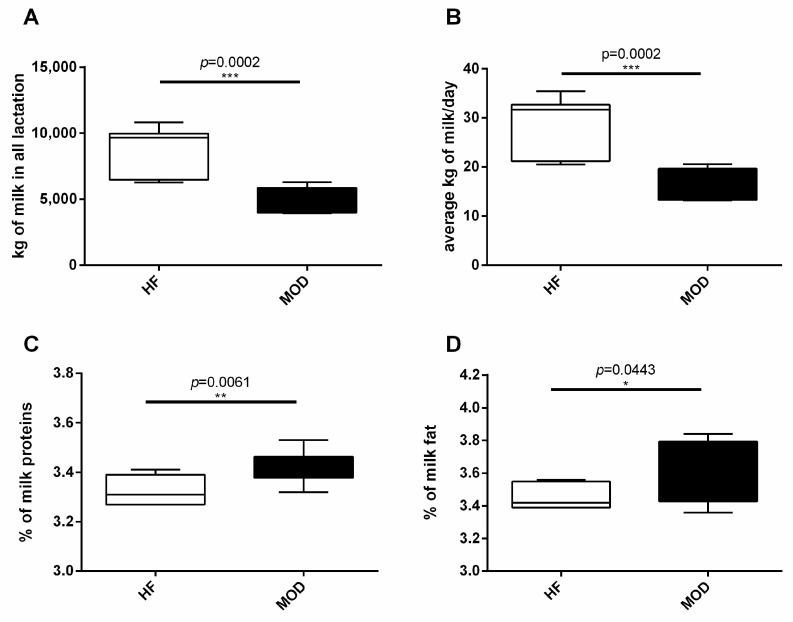
Milk production data. Daily milk production and the content of proteins and fat in milk were registered for each animal under study: (**A**) total milk production during the analyzed lactation (kg); (**B**) daily milk yield average (kg); (**C**) milk protein content (%); (**D**) milk fat content (%). The number of asterisks (*, **, ***) indicates the level of statistical significance.

The MOD animals included in our study showed a significantly lower milk production compared to HF, both in terms of daily average milk yield (HF = 29.29 kg vs. MOD = 16.03 kg, *p* = 0.0002) and total milk production (HF = 8940 kg vs. MOD = 4851, *p* = 0.0002) (Figure 1A,B); nevertheless, their milk demonstrated higher quality when compared to the HF group. In particular, the percentages of milk protein (HF = 3.33% vs. MOD = 3.42%) and fat (HF = 3.46% vs. MOD = 3.62%) were significantly higher in MOD compared to HF (*p* = 0.0061 and, *p* = 0.0443, respectively), as shown in Figure 1C,D.

### 3.2. Bacteriological Analyses

**Figure 2:**
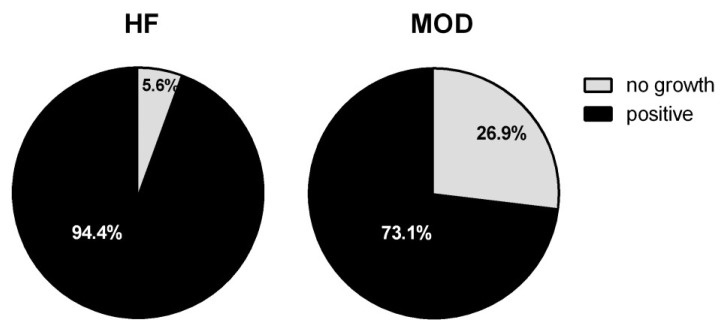
Quarters with negative bacterial growth. Bacteriological examination was conducted on all milk quarter samples. After cultural analysis, the milk samples that did not show bacterial growth were defined as “no growth”, whereas those with bacterial colonies were defined as “positive” for bacterial growth (Fisher’s test; n HF = 72 samples, n MOD = 96 samples).

In the bacteriological analyses, MOD cows showed a significantly lower number of culture-positive quarters compared to HF (*p* < 0.0001; Figure 2). The most frequent bacteria were non-aureus *Staphylococcus* and *Mammaliicoccus* (NAS) in both cow groups, although with a different prevalence: 47.2% of quarters in HF vs. 21.5% in MOD (Supplementary Table S1). Interestingly, we found three quarters with positive results for *S. aureus* in MOD, which spontaneously resolved within a month (Supplementary Table S1). Concerning the somatic cell counts (SCC), no significant difference was observed at any time point between the two experimental groups (HF vs. MOD). However, we interestingly observed a significant correlation between SCC and the type of infection (*p* = 0.01), whereas no such correlation was found with the number of isolated colonies. The bacteria that seemed to highly increase SCC were *S. aureus* and Gram-negative bacteria. We also found culture-negative quarters with elevated SCC values.

### 3.3. Similar E. coli Bacterial Killing Activity of the Milk Was Shown by Cows with Allegedly Low and High Susceptibility to Mastitis

**Figure 3:**
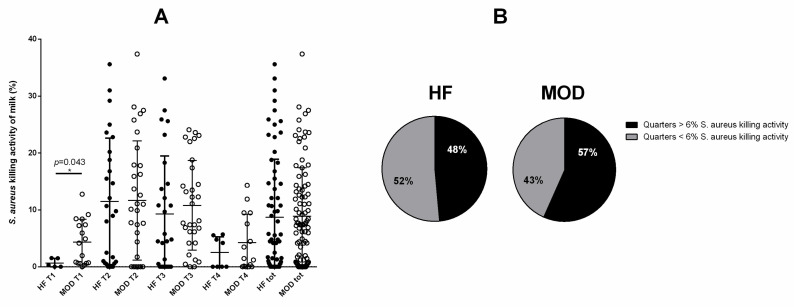
*S. aureus* killing activity. (**A**) The killing activity was measured by flow cytometry as a cumulative percentage of dead and subvital bacteria after incubation of log-phase *S. aureus* in skim acellular milk (1:2 in BHI medium) compared to the same bacteria incubated only in BHI medium (control). (**B**) Defining 6% as the cut-off of *S. aureus* killing ability, we compared the % of quarters in both animal groups (HF and MOD) with a killing activity higher or lower compared to the cut-off. The number of asterisks (*) indicates the level of statistical significance.

Skim and acellular quarter milk samples were analyzed by flow cytometry to measure the bacterial killing activity. Some samples had either very low or very high activity. In particular, the range of *E. coli* killing activity varied between 0 and 32.4% in MOD, compared to 0 and 13.3% in HF cows. Given the number of events acquired by the flow cytometer (10,000), we defined as a cut-off an *E. coli* killing ability > 6%. No differences were evident among MOD and HF groups in the number of quarters with an *E. coli* killing activity > 6% (Figure 3). The *E. coli* killing activity was negatively correlated with days in milk (DIM) (*p* < 0.0001) (Table 1).

### 3.4. A Tendency to Greater S. aureus Killing Activity of the Milk Was Shown by Cows with Allegedly Low Susceptibility to Mastitis

We evaluated the *S. aureus* killing activity using flow cytometry. The range of *S. aureus* killing activity varied between 0 and 37.4% in MOD, compared to 0 and 35.6% in HF cows. The *S. aureus* killing activity of the MOD group resulted in significantly stronger at T1 compared to the HF group (*p* = 0.043; Figure 3A). No significant differences were observed at other time points between the two experimental groups. Given the number of acquired events by the flow cytometer (10,000), we also defined as cut-off an *S. aureus* killing ability > 6%. The MOD group showed a higher number of quarter samples beyond cut-off compared to the HF group (55% vs. 44%; *p* = 0.171) (Figure 3B). Also, the *S. aureus* killing activity resulted negatively correlated with DIM (*p* < 0.0001) (Table 1). Finally, MOD and HF samples were matched for bacterial growth: culture-positive MOD samples showed no significant difference compared to culture-positive HF samples. The same held true of culture-negative MOD and HF samples (Supplementary Figure S3).

### 3.5. The Killing Activity of Cow Milk Is More Effective against S. aureus Than E. coli

**Figure 4:**
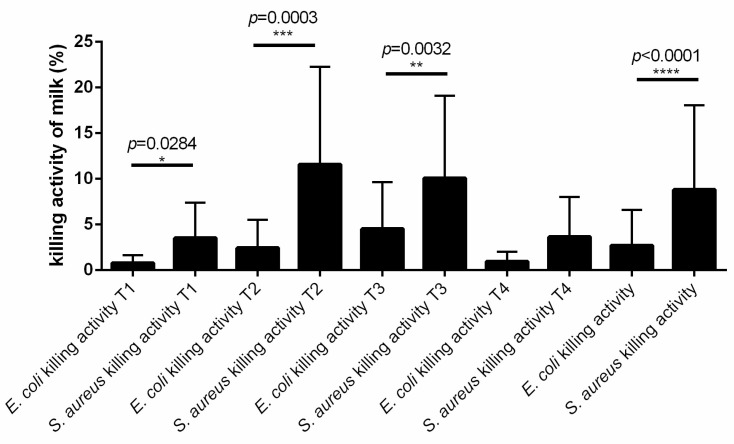
Milk *S. aureus* and *E. coli* killing activity at different time points. The killing activity was measured by flow cytometry as cumulative percentage of dead and subvital bacteria after incubation of log-phase *S. aureus* or *E. coli* in skim acellular milk (1:2 in BHI medium) compared to the same bacteria incubated only in BHI medium (control) at different time points: T1 (dry-off), T2 (1 day postpartum), T3 (7–10 days postpartum), and T4 (30 days postpartum). The number of asterisks (*, **, ***, ****) indicates the level of statistical significance.

Because in both experimental groups, the milk killing activity seemed more effective against *S. aureus* than *E. coli*, we compared the killing activity of all milk samples at all the time points against *S. aureus* and *E. coli.* We observed that skim acellular milk had a higher killing activity against *S. aureus* compared to *E. coli* at all time points, except T4, and globally (sum of samples at all time points, *p* < 0.0001; Figure 4).

### 3.6. NAGase Activity Is Involved in the Control of S. aureus and It Is Stimulated by Milk Microbiota or IMI

**Figure 5:**
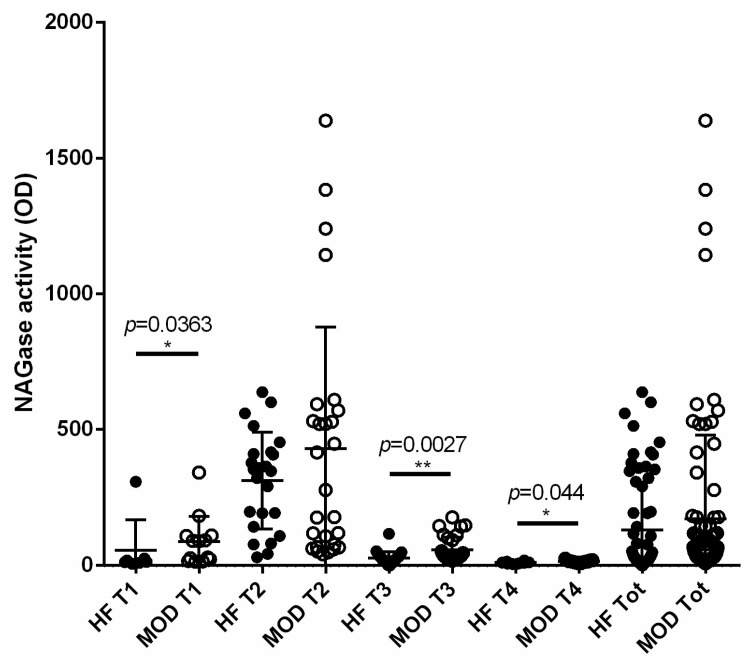
NAGase activity of milk at different time points. NAGase activity was measured using a fluorescence-based procedure on quarter milk samples of HF and MOD cows. NAGase activity of HF and MOD cows is shown at different time points (T1 to T4). The last two groups depict all the milk samples of the whole study. The number of asterisks (* to **) indicates the level of statistical significance.

In order to find a correlation between bacterial killing and innate immunity, we investigated NAGase in the same samples. We observed a significant difference between the two groups of samples at T1 (*p* = 0.0363), T3 (*p* = 0.0027), and T4 (*p* = 0.044; Figure 5) with a greater activity in the MOD group with respect to HF at these time points. A correlation analysis demonstrated a significant negative correlation with the time point and DIM (both *p* < 0.0001) (Table 2). Interestingly we also observed a positive correlation between NAGase activity and *S. aureus* killing activity (*p* < 0.0001), and also a correlation between NAGase activity and the type of bacteria isolated in milk and colostrum samples (*p* = 0.0001) (Table 2). Moreover, a significant correlation between NAGase and the killing of *E.coli* was observed in both culture-negative and low-SCC samples (*p* = 0.01). As for *S. aureus*, such a correlation was actually lower in culture-negative samples (*p* = 0.0485), but dramatically higher in the low-SCC ones (*p* < 0.0001). Accordingly, we confirmed a global positive correlation between NAGase activity and SCC (*p* < 0.0001) (Table 2).

Interestingly, the highly significant positive correlation between NAGase activity and *S. aureus* killing activity observed in the analysis of all the quarter samples, dropped when analyzing the culture-negative ones (*p* = 0.0485; Table 3); however, this correlation remained high in quarter samples with SCC < 200,000 (*p* < 0.0001; Table 4).

**Figure 6:**
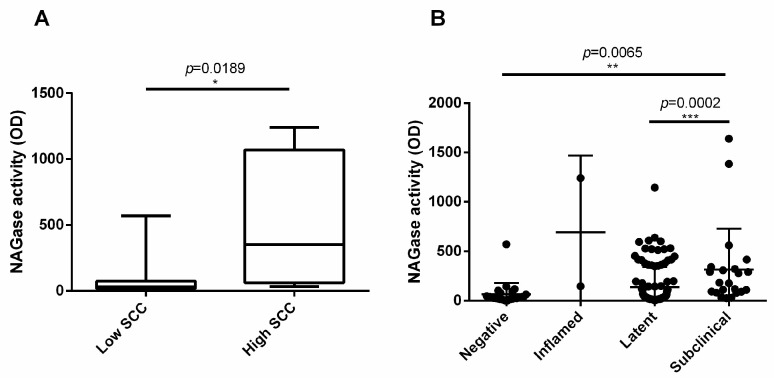
NAGase activity of milk. NAGase activity was measured using a fluorescence-based procedure on quarter milk samples. (**A**) NAGase activity was analyzed in samples with negative bacterial growth and with a SCC < 200,000 compared to negative bacterial growth samples with SCC > 200,000. (**B**) NAGase activity was evaluated in quarter samples divided into negative, inflamed, latent, and subclinical. Inflamed quarters were not analyzed because of their paucity. The number of asterisks (*, **, ***) indicates the level of statistical significance.

Furthermore, NAGase activity resulted significantly higher in culture-negative quarter samples with SCC > 200,000 compared to culture-negative samples with SCC < 200,000 (*p* = 0.0189; Figure 6A). Finally, we classified the quarter samples as negative, inflamed, latent, and subclinical, as described in the Section 2 [20]. Interestingly, the NAGase activity significantly and gradually increased from negative to subclinical samples (*p* = 0.0002; Figure 6B).

## 4. Discussion

Intramammary infections of cows are still one of the biggest issues in the dairy industry, leading to important economic losses and extensive drug usage. Subclinical mastitis cases are difficult to diagnose and cause a reduction in both milk yield and quality. To overcome this problem, a new approach to disease control is badly needed. Recent studies demonstrated that more effective innate immune responses can be found in autochthonous cattle when compared to Holstein cows [19]. Therefore, autochthonous breeds could serve as a valid model to investigate the molecular mechanisms underlying the more efficient response of the mammary gland in the antimicrobial fight. In this respect, our study was not intended to demonstrate the extent of antibacterial control in the milk of autochthonous and cosmopolitan dairy cattle breeds. Also, the concept of mastitis-resistant breeds must be viewed with caution, since, even among HF cows, there are mastitis-resistant animals, which further compounds this critical issue. Thus, our sampling scheme aimed to validate our working hypotheses in genetically different dairy cattle, thus ruling out a possible breed-related experimental bias. As expected, MOD cows, reared on the same farm as HF, were less susceptible to IMI in terms of the number of quarters with negative bacteriological growth. According to circumstantial evidence, one MOD cow with two *S. aureus* culture-positive quarters underwent spontaneous clearance of the pathogen within 30 days without any therapeutical intervention, which is absolutely unusual in cosmopolitan HF dairy cows. Next, we investigated the different responses of the bovine mammary gland to Gram-positive and Gram-negative pathogens. Specifically, we investigated the bacterial killing activity of colostrum and milk against both a Gram-positive pathogen (*S. aureus*) and a Gram-negative one (*E. coli*). Our flow cytometry data were validated on the basis of the above-mentioned internal controls of the kit (see Section 2.4). As a caveat, the number of cows at both T1 and T4 was lower compared to other time points and less than optimal for comparative purposes. Beyond this regrettable feature, the killing activity of mammary secretum was shown to be more pronounced against *S. aureus* compared to *E. coli*, suggesting that Gram-positive mastitis agents could be preferentially controlled by humoral components in colostrum and milk. Most importantly, our results show that killing is an innate property of healthy, culture-negative bovine milk, which is modulated by subsequent IMIs (see the Section 3) and, probably, by the milk bacterial microbiota and minor/dubious pathogens like NASM. On the other hand, Gram-negative bacteria induce a very strong inflammatory response in the udder, stimulating mammary epithelial cells and leading to the recruitment of immune cells, mainly phagocytes [29]. Conversely, Gram-positive bacteria poorly stimulate instead the mammary epithelial cells, while strongly activating professional innate immune cells, such as macrophages. The killing activity against *S. aureus* was higher in the MOD group at T1; MOD cows also resulted in more resistance to IMI, as indicated by the significantly higher number of culture-negative quarter samples compared to the HF group. The aforementioned results demand a meticulous examination of the humoral effector mechanisms underlying the preferential killing of *S. aureus* observed in several colostrum/milk samples. Overall, *S. aureus* poorly induces antibacterial peptides in mammary epithelial cells [30]. Moreover, even if antibacterial peptides were induced by a previous IMI, we should keep in mind that *S. aureus* has developed powerful mechanisms of resistance to such peptides, as previously shown in pediatric patients [31]. This undoubtedly raises questions about their potential role in our bacterial killing assay. Furthermore, the milk microbiota [32] represents a source of effective bacteriocins [33], reputedly active on biofilm-positive *S. aureus* strains [34]. Accordingly, recognized variations in the milk microbiota during different phases of lactation, as well as between autochthonous and cosmopolitan cattle breeds [20], could be responsible for different bacteriocin profiles and associated antibacterial activity. As for the antibody response in milk, contradictory and diverging results have been reported. Firstly, it is important to emphasize that a lytic, complement-mediated pathway for bacterial killing is unlikely to occur, given the limited availability of C1q for such activity in the milk from healthy quarters. Additionally, the milk environment is endowed with a powerful anti-phagocyte activity, hampering the activity of common opsonins like antibodies [35]. On the other hand, there are indications of a possible role exerted by antibodies of the IgA isotype. Thus, *S. aureus*-specific vaccine-induced IgA antibodies in milk successfully suppressed the multiplication of the pathogen in infected bovine udders [36]. Since IgA does not activate the classical complement pathway, other effector mechanisms should be implied; among these, a modulation of macrophage antibacterial activity by IgA-*S. aureus* immunocomplexes binding to surface CD89 is quite possible [37]. However, these mechanisms are not relevant to our working scenario, in which bacterial killing was performed by acellular samples. Moreover, natural antibodies (Nab) of the IgG1, IgG2, IgM, and IgA isotypes exhibit binding capabilities. Nevertheless, it is unlikely that Nab performs bacterial killing in acellular samples, due to the aforementioned reasons.

Our findings also indicated a significantly higher NAGase activity in quarter samples with positive bacterial growth compared to culture-negative samples. NAGase was shown to act as an agonist in our killing assay, due to its target specificity (cell wall peptidoglycan abundantly expressed in *S. aureus*), and to the strong, consistent, and significant correlation observed with bacterial killing in the investigated quarters. Indeed, NAGase is a component of the innate immune system, expressed by a variety of cells. In healthy quarters, mammary gland secretory cells are likely to be the primary source of this enzyme in milk. However, during an inflammatory process, most of the NAGase activity in milk probably derives from blood serum, macrophages, and neutrophils. Accordingly, clinical mastitis cases exhibit higher milk NAGase levels compared to subclinical ones [38]. In line with Selva Rani’s study (see references), we also observed a gradual, significant increase in the NAGase activity from negative to subclinical quarter samples.

Since high levels of killing were observed in many samples with low levels of SCC, it should be stressed that even in these samples a correlation between bacterial killing and NAGase activity was found. This correlation was notably weaker in culture-negative samples overall, providing compelling evidence for the involvement of non-pathogenic bacteria in the modulation of NAGase levels in mammary epithelial cells of healthy quarters. This scenario is also in agreement with the strong correlation between NAGase and SCC in all the analyzed samples. Lysozyme is a further possible agonist of bacterial killing, with additive/synergistic activity against peptidoglycan in the presence of NAGase. Interestingly, there is strong evidence that cosmopolitan HF dairy cattle express little if any lysozyme in serum, let alone in milk, as opposed to autochthonous cattle breeds [39]. In addition, lipoteichoic acid (LTA) and peptidoglycan are present in milk, although at lower concentrations compared to plasma [40]. Furthermore, PTX3 could contribute to the killing activity. PTX3 is a long pentraxin, characterized by a pentameric structure and showing both opsonizing functions and immune response modulation activity [41]. PTX3 represents a soluble pattern recognition receptor produced by several types of immune and non-immune cells. PTX3 was demonstrated to bind certain pathogens and complement sub-units. Different studies indicate PTX3 as a specific diagnostic and prognostic marker in opportunistic infections, including those caused by *S. aureus* [42,43,44,45]. Moreover, PTX3 has been shown to be upregulated in *S. aureus* mammary infections in both goats and cattle. However, its specific role in the mammary gland defense and the possible mechanism of bacterial inhibition remains unknown [42,46,47,48,49]. Additionally, peptidoglycan recognition proteins (PGLYRP1-PGLYRP4) could play a role in the killing activity of colostrum and milk against bacteria [50,51]. These molecules belong to the PRR superfamily and are highly conserved from insects to mammalians. They can kill bacteria both directly by interfering with their cell walls and indirectly by modulating inflammation and immune response [47,48]. Lastly, the potential role of milk lactoferrin should also be considered, given the demonstrated susceptibility of *S. aureus* in growth inhibition assays [39].

Beyond the possible effector mechanisms of bacterial killing, this property of bovine milk will have to be investigated with respect to its biological significance, i.e., the correlation between the extent of killing observed by flow cytometry and fundamental bacterial properties like % vitality in bacteriological media, secretion of toxins, biofilm formation, and intracellular persistence. This way, the real meaning of bacterial killing and its implications for mastitis control could be evaluated on a robust basis toward an improvement of the present diagnostic and research-oriented assays for bovine mastitis.

## 5. Conclusions

Bacterial killing observed in acellular bovine colostrum and milk samples is preferentially exerted against *S. aureus* when compared to *E. coli*. This activity is attributed to a combination of innate immune mechanisms, including the activity of NAGase and others that need to be characterized with further investigation. Variations in bacterial killing may occur due to factors such as lactation period, microbiological and cellular (SCC) status of the samples, and the specific breed of dairy cattle under investigation. These findings highlight a *S. aureus* killing mechanism, with potential implications for a deeper understanding of the udder’s immune defenses. These insights could also be conducive to the development of new adjuvants for vaccines against mastitis or new immunotherapeutic protocols to protect the mammary gland. Further studies will hopefully identify the humoral components responsible for bacterial killing in milk and their modulation, toward the possible development of new effective drugs for mastitis control.

## Figures and Tables

**Table 1:** Correlation among bacterial killing activity and other parameters.

Parameter	Correlation	*p* Value	R Spearman
*E. coli* killing activity	DIM	<0.0001 ****	−0.36
	Time point ^§^	0.0365 *	0.15
	Type of IMI ^§§^	0.0015 **	0.24
	NAGase activity	0.018 *	0.18
	*S. aureus* killing activity	<0.0001 ****	0.34
*S. aureus* killing activity	DIM	<0.0001 ****	−0.45
	*E. coli* killing activity	<0.0001 ****	0.34
	NAGase activity	<0.0001 ****	0.44
	Etiology of IMI	0.0027 **	0.25

The correlation among the different parameters under study was investigated by the two-tailed rho tests of Spearman. ^§^ Time points: T1 = dry off, T2 = 1 day postpartum, T3 = 7–10 days postpartum, T4 = 30 days postpartum. §§ The type of IMI includes no bacterial growth, NSR (not significant result), non-aureus Staphylococci and Mammaliicocci (NASM), *S. aureus*, *S. dysgalactiae*, *A. viridans*, *Proteus* spp., *E. faecalis*, *S. xylosus*, *Corynebacterium* sp., *C. bovis*, *C. stationis*, *Streptococcus* sp., *S. chromogenes*, *S. haemoliticus*, *S. succinius*, *L. paracasei*, *A. johnsonii*, *A. iwoffii*, *Bacillus* sp., *B. subtilis*. The number of asterisks (*, **, ****) indicates the level of statistical significance.

**Table 2:** Correlation among NAGase activity and other parameters in all the quarter samples under study.

Parameter	Correlation	*p* Value	R Spearman
NAGase activity	Time point ^§^	<0.0001 ****	−0.54
	DIM	<0.0001 ****	−0.73
	SCC	<0.0001 ****	0.62
	Type of IMI ^§§^	0.0001 ***	0.30
	*S. aureus* killing activity	<0.0001 ****	0.44

The correlation among the different parameters under study was investigated by the two-tailed rho tests of Spearman. ^§^ Time points: T1 = dry off, T2 = 1 day postpartum, T3 = 7–10 days postpartum, T4 = 30 days postpartum. ^§§^ The type of IMI includes no bacterial growth, NSR, NASM, *S. aureus*, *S. dysgalactiae*, *A. viridans*, *Proteus* spp., *E. faecalis*, *S. xylosus*, *Corynebacterium* sp., *C. bovis*, *C. stationis*, *Streptococcus* sp., *S. chromogenes*, *S. haemoliticus*, *S. succinius*, *L. paracasei*, *A. johnsonii*, *A. iwoffii*, *Bacillus* sp., *B. subtilis*. The number of asterisks (*** to ****) indicates the level of statistical significance.

**Table 3:** Correlation among NAGase activity and other parameters in quarters with negative bacterial growth.

Parameter	Correlation	*p* Value	R Spearman
NAGase activity	Time point ^§^	<0.0001 ****	−0.68
	DIM	<0.0001 ****	−0.87
	SCC	0.0048 **	0.52
	*E. coli* killing activity	0.0111 *	0.47
	*S. aureus* killing activity	<0.0485 *	0.40

The correlation among the different parameters under study was investigated by the two-tailed rho tests of Spearman. ^§^ Time points: T1 = dry off, T2 = 1 day postpartum, T3 = 7–10 days postpartum, T4 = 30 days postpartum. The number of asterisks (*, **, ****) indicates the level of statistical significance.

**Table 4:** Correlation among NAGase activity and other parameters in low SCC quarter samples (SCC < 200,000).

Parameter	Correlation	*p* Value	R Spearman
NAGase activity	Time point ^§^	<0.0001 ****	−0.52
	DIM	<0.0001 ****	−0.85
	SCC Type of IMI ^§§^	<0.0001 **** 0.0007 ***	0.60 0.29
	*E. coli* killing activity	0.0177 *	0.21
	*S. aureus* killing activity	<0.0001 ****	0.60

The correlation among the different parameters under study was investigated by the two-tailed rho tests of Spearman. ^§^ Time points: T1 = dry off, T2 = 1 day postpartum, T3 = 7–10 days postpartum, T4 = 30 days postpartum. The number of asterisks (*, ***, ****) indicates the level of statistical significance. ^§§^ The type of IMI includes no bacterial growth, NSR, NASM, *S. aureus*, *S. dysgalactiae*, *A. viridans*, *Proteus* spp., *E. faecalis*, *S. xylosus*, *Corynebacterium* sp., *C. bovis*, *C. stationis*, *Streptococcus* sp., *S. chromogenes*, *S. haemoliticus*, *S. succinius*, *L. paracasei*, *A. johnsonii*, *A. iwoffii*, *Bacillus* sp., *B. subtilis.*

## Data Availability

Data are contained within the article and Supplementary Materials.

## Supplementary Materials

The following supporting information can be downloaded at: https://www.mdpi.com/article/10.3390/vetsci11040166/s1, Figure S1: FACS gating strategy for the analysis of killing activity of milk; Table S1: Type of IMI, Figure S2: G*power analysis of sample size and statistical power; Figure S3: Bacterial killing activity in culture negative and positive quarters of MOD and HF.
