# Supplementary material for: Different Immune Control of Gram-Positive and Gram-Negative Mammary Infections in Dairy Cows
Source: Vet Sci. 2024 Apr 6;11(4):166. doi: 10.3390/vetsci11040166 (PMC11054201; doi:10.3390/vetsci11040166)
Supplement: Supplementary file 1 [file vetsci-11-00166-s001.zip › vetsci-2784697-supplementary.pdf]

SUPPLEMENTARY MATERIAL

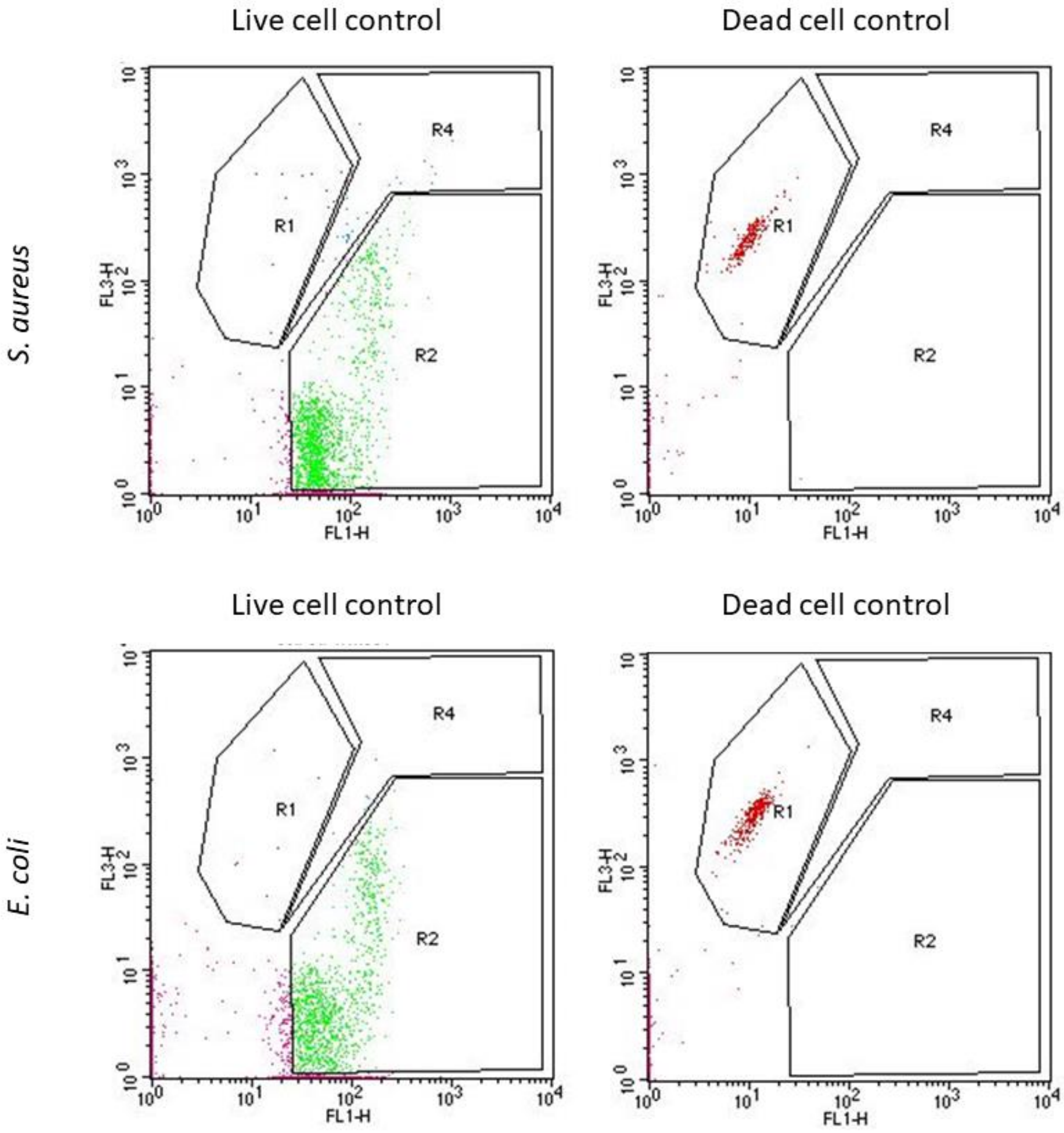

Supplementary Figure S1. FACS gating strategy for the analysis of killing activity of milk.

Bacterial cells were stained with both Cyto 9 (x axis, FL1) and Propidium iodide (y axis, FL3) , staining live and dead bacterial cells in the green and red fluorescence channels, respectively, and gated by a combination of forward and side scatter. Bacterial viability was analyzed in a red x green cytogram, which allowed for discrimination between dead/live/subvital cells. In particular, live cells can be seen in Region 2 (R2), dead cells in R1 and the subvital ones in R4. Log phase *E. coli* and *S. aureus* grown in BHI medium for 2 hours (live cell control); Log phase *E. coli* and *S. aureus* in BHI medium for 2 hours, resuspended in 70% isopropyl alcohol, and kept at room temperature for 60 minutes (dead cell control).

Supplementary Table S1. Type of IMI

Type of IMI	N of quarters MOD	N of quarters HF
NSR* (not significant result)	40	17
NASM (Non-aureus *Staphylococci* and *Mammaliicocci*)	12	31
S. aureus	3	0
S. dysgalactiae	0	1
A. viridans	0	2
Proteus spp.	0	4
E. faecalis	0	1
S. xylosus	2	0
Corynebacterium sp.	0	1
C. bovis	2	0
C. stationis	0	1
Streptococcus sp.	1	2
S. chromogenes	7	3
S. haemoliticus	0	1
S. succinius	0	1
L. paracasei	1	0
A. johnsonii	1	0
A. iwoffii	0	2
Bacillus sp.	0	3
B. subtilis	0	1

- NSR means that the isolated bacteria were not relevant for IMI.

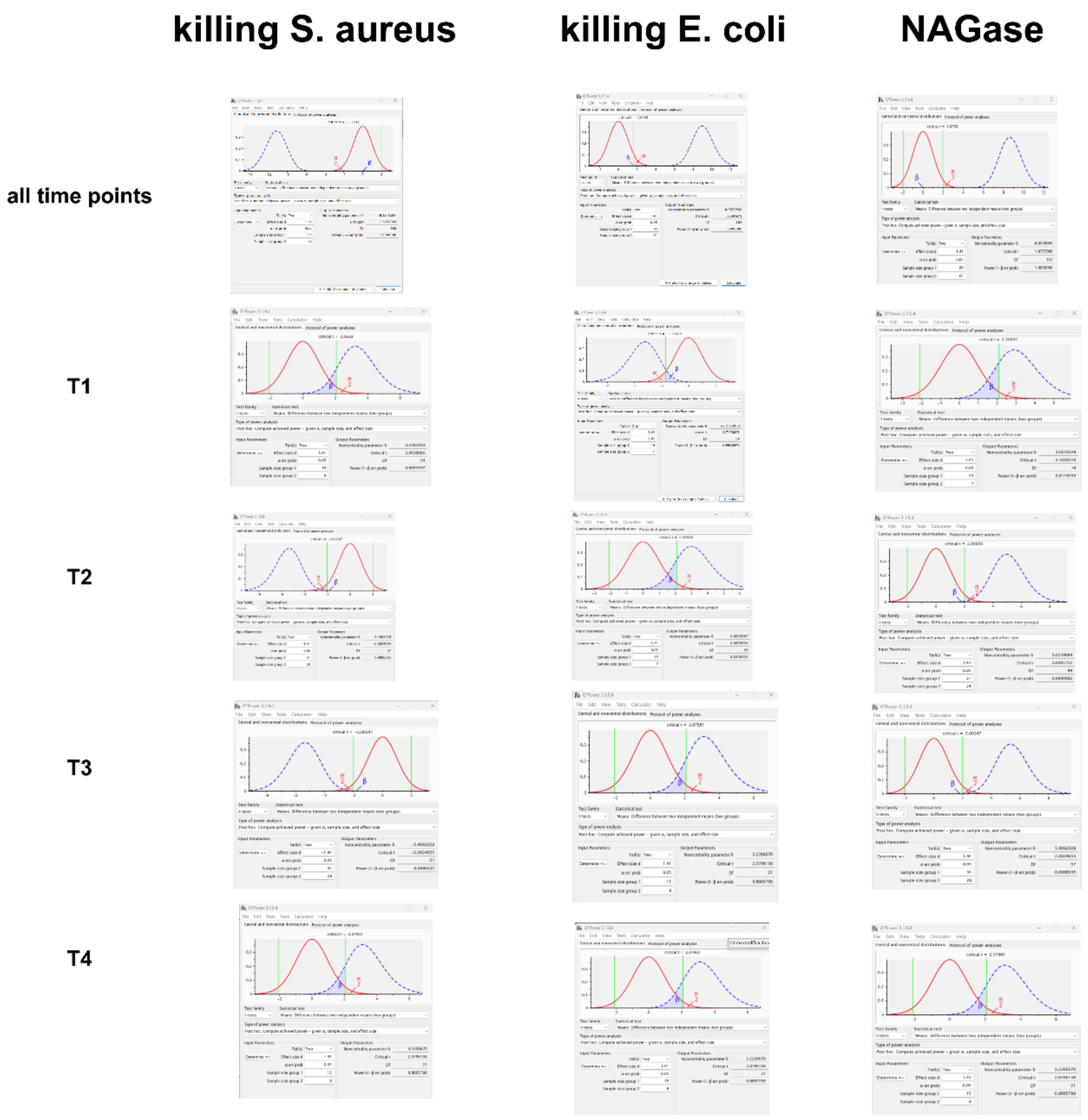

Supplementary Figure S2. G*power analysis of sample size and statistical power.

The figure shows the screen shots of the G*power software analysis (G*power software version 3.1.9.4 (Franz Faul, Universitat Kiel, Germany) used to evaluate the statistical power of our analysis based on the sample size used.

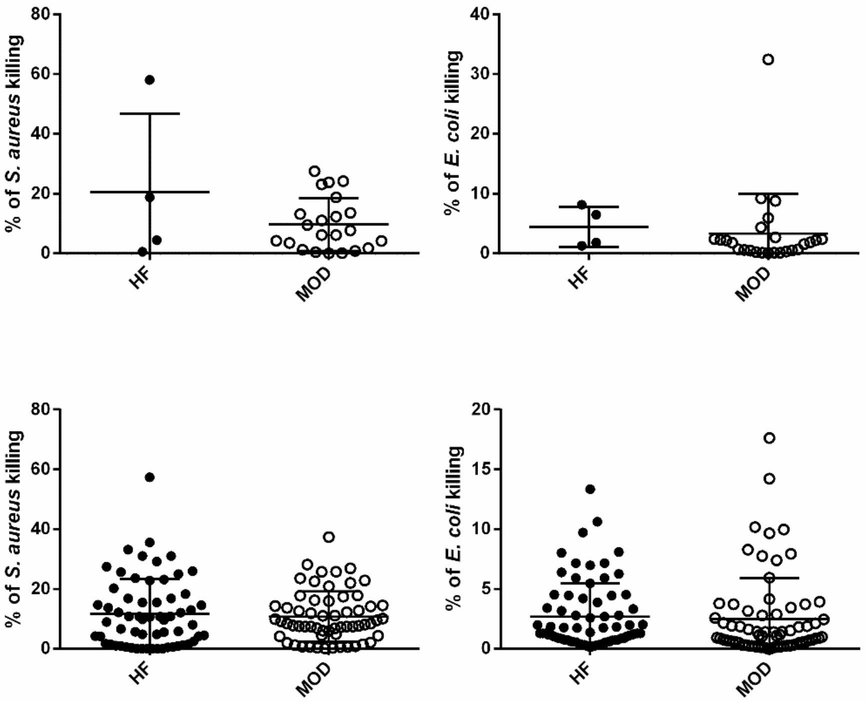

Supplementary Figure S3. Bacterial killing activity in culture negative and positive quarters of MOD and HF.

The figure shows the comparison of the *S. aureus* and *E. coli* killing activity of culture negative and positive quarters in the two cattle breeds under study.
